# Opinion attribution improves motivation to exchange subjective opinions with humanoid robots

**DOI:** 10.3389/frobt.2024.1175879

**Published:** 2024-02-19

**Authors:** Takahisa Uchida, Takashi Minato, Hiroshi Ishiguro

**Affiliations:** ^1^ Graduate School of Engineering Science, Osaka University, Osaka, Japan; ^2^ Advanced Telecommunications Research Institute International (ATR), Kyoto, Japan; ^3^ RIKEN, Kyoto, Japan

**Keywords:** subjective opinion, opinion attribution, motivation, dialogue robot, android, humanoid robot

## Abstract

In recent years, the development of robots that can engage in non-task-oriented dialogue with people, such as chat, has received increasing attention. This study aims to clarify the factors that improve the user’s willingness to talk with robots in non-task oriented dialogues (e.g., chat). A previous study reported that exchanging subjective opinions makes such dialogue enjoyable and enthusiastic. In some cases, however, the robot’s subjective opinions are not realistic, i.e., the user believes the robot does not have opinions, thus we cannot attribute the opinion to the robot. For example, if a robot says that alcohol tastes good, it may be difficult to imagine the robot having such an opinion. In this case, the user’s motivation to exchange opinions may decrease. In this study, we hypothesize that regardless of the type of robot, opinion attribution affects the user’s motivation to exchange opinions with humanoid robots. We examined the effect by preparing various opinions of two kinds of humanoid robots. The experimental result suggests that not only the users’ interest in the topic but also the attribution of the subjective opinions to them influence their motivation to exchange opinions. Another analysis revealed that the android significantly increased the motivation when they are interested in the topic and do not attribute opinions, while the small robot significantly increased it when not interested and attributed opinions. In situations where there are opinions that cannot be attributed to humanoid robots, the result that androids are more motivating when users have the interests even if opinions are not attributed can indicate the usefulness of androids.

## 1 Introduction

This study aims to clarify the factors that improve the user’s willingness to talk with robots in non-task oriented dialogues (e.g., chat). They are expected to play an important role in various applications, such as communication support for elderly people (Su et al., 2017). Additionally, in situations where the importance of communication is addressed, there is a great social demand for dialogue robots.

The spoken dialogue systems required for these robots can be classified into two types (Chen et al., 2017): task-oriented (e.g., (Williams and Young, 2007)) and non-task-oriented (e.g., (Wallace, 2009)) dialogue. In a task-oriented dialogue, the policy of the dialogue strategy is to achieve a specific goal, such as finding products (Yan et al., 2017) or seat reservation (Seneff and Polifroni, 2000). However, in non-task-oriented dialogues, the goal of the dialogue is to continue the dialogue by stimulating the motivation of the user. Hence, many studies have aimed at generating linguistically connected utterances. A famous non-task-oriented dialogue system ELIZA (Weizenbaum, 1966) has a dialogue database and response patterns. They enable it to continue a dialogue using parts of the user’s speech without limiting the topic. Recently, some studies have aimed to generate system utterances with more natural connections by selecting ones using human-human dialogue data (Huang et al., 2022). Other studies have focused on dialogue breakdown detection technology aiming to avoid speech without linguistic connections (Higashinaka et al., 2016). While linguistic consistency is an essential part of dialogue, stimulating the user’s motivation to talk is also necessary for non-task-oriented dialogue. Even if the utterances are linguistically correct, the user will soon get bored if they do not have the motivation to talk to the system. Therefore, to continue a dialogue, the system should stimulate the user’s desire to interact.

One method to stimulate the user’s motivation is to recognize the user’s interest and talk about them. Some studies have proposed methods to estimate the current level of interest based on various information (e.g., (Hirayama et al., 2011)). For estimating preferences, Uchida et al. developed a dialogue system that estimates the user’s preferences (Uchida et al., 2021). Other methods estimate user interests not only by linguistic information (Chen et al., 2022) but also by combining linguistic and non-linguistic information (Yu et al., 2019).

A previous study reported that exchanging subjective opinions makes chats enjoyable and enthusiastic (Tokuhisa and Terashima, 2009). The exchange of subjective opinions can be a self-disclosure and is a factor that plays an important role in the process of intimacy in interpersonal relationships (Altman and Taylor, 1973). The alignment of internal states in communication leads to the building of mutual trust through the enhancement of the predictability of the participants’ action choices (Katagiri et al., 2013). Yuan et al. developed the system that accomplish human-robot mental alignment (Yuan et al., 2022) in the context of explainable artificial intelligence (XAI) (Edmonds et al., 2019). Therefore, the exchange of subjective opinions is effective for intimacy with the interlocutor and can be a means of stimulating motivation for dialogue.

In this study, we define subjective opinion as “an evaluation made by an individual on a target.” For example, if the robot evaluates pasta as tasty, the robot’s subjective opinion is “the pasta is tasty.” From the previous studies mentioned above, we deduce that it can be effective for robots to express their subjective opinion to make the user feel enjoyable and enthusiastic in dialogue. However, another study has stated that people hardly attribute subjective experiences related to value (good or bad) to robots (Sytsma and Machery, 2010). Therefore, even if the robot states that food tastes good, its user is unlikely to attribute the experience of eating the food to it and to find that the robot actually has the opinion. Thus, in some cases, the robot’s subjective opinions are not realistic, i.e., the user does not feel the robot has its own opinions. This study refers to this phenomenon as “the user does not attribute subjective opinions to the robot.” We speculate that this phenomenon may reduce the user’s motivation to exchange opinions with the robot.

This study hypothesizes that regardless of the type of robot, opinion attribution affects the user’s motivation to exchange opinions with robots. For example, if a robot says that alcohol tastes good, it may be difficult to imagine the robot having such an opinion. On such topics, the user’s motivation to exchange opinions is likely to decrease. The contribution of this study is to show that opinion attribution is important for exchanging opinions with robots and to generalize the findings of previous studies to communication robots. Furthermore, people are willing to differently interact with different types of agents (Uchida et al., 2017a; 2020). We discuss a strategy to select the type of communication robots for various classes of dialogues and embodiment of the dialogue robots by considering the robot’s appearance and its impact on dialogue. In summary, the contributions of this study are the following:

• To clarify the influence of opinion attribution to humanoid robots on users’ motivation to exchange opinions with them.
• To clarify the topics that improve the user’s motivation to exchange opinions with each type of humanoid robot.

## 2 Related works

Our study is related to a cognitive phenomenon: mental state attribution. It means “the cognitive capacity to reflect upon one’s own and other persons” mental states such as beliefs, desires, feelings and intentions (Brüne et al., 2007)” and helps us to understand the others (Dennett, 1989; Mithen, 1998; Epley et al., 2007; Heider, 2013). Many previous studies reported that more human-like appearance increases the degree of mental state attribution to robots (Krach et al., 2008; Broadbent et al., 2013; Takahashi et al., 2014; Martini et al., 2016; 2015; Xu and Sar, 2018; Banks, 2020; Manzi et al., 2020). On the other hand, our study specifically focuses on the effect of opinion attribution on users’ motivation to exchange opinions with humanoid robots. Another previous study investigated the effect of the robot’s appearance on the users’ cooperative attitude toward it (Goetz et al., 2003). While it has investigated the cooperative attitudes of users in a task, this study focuses on the speech content of the robot.

In our previous study, we investigated the effect of opinion attribution on motivation to talk with a human-like android (Uchida et al., 2019). We asked participants to evaluate whether they had attributed opinions to it and their motivation to talk with it regarding a variety of opinions (e.g., the taste of food and the performance of a computer). As a result, we clarified the user’s interest in the android’s opinions and the attribution of the subjective opinions to it influence their motivation for dialogue. Because we investigated the effect of opinion attribution on the dialogue motivation using only the android in the previous study, this result could not be generalized to all dialogue robots. In addition, our previous study used the question “whether or not you want to dialogue” to evaluate it (Uchida et al., 2019). Since the dialogue can contain various factors, it is not clear whether the opinion attribution has an influence on the exchange of subjective opinions.

Recently, there have been many studies on artificial systems for engaging users in conversations, such as chatting, aiming to continue the dialogue itself (i.e., non-task-oriented dialogue). Contributing to this trend are developments in natural language processing (e.g., GPT4 (OpenAI, 2023)) and speech recognition technology (Baevski et al., 2020), which has shown tremendous progress in recognizing user speech. However, long-term non-task-oriented dialogues with the users such as human-to-human dialogues have not been realized yet. For such long-term dialogues, it is important to exchange subjective opinions (Tokuhisa and Terashima, 2009). However, it has been suggested that when an android which has human-like appearance utters subjective opinions, if that opinion cannot be attributed to it, then the user’s motivation to talk decreases (Uchida et al., 2019). In this study, we examined whether this finding is applicable to other humanoid robots and how opinion attribution and motivation to exchange opinions differ depending on the type of robot.

## 3 Materials and methods

We adopted the same experimental procedure as the previous study (Uchida et al., 2019) using an android (ERICA (Glas et al., 2016)) and a small robot (SOTA^1^). We used an android, which is a humanoid robot, that closely resembles a human in appearance, and a small robot that has a machine-like appearance. The hypothesis of this experiment is that, regardless of the type of robot, attributing opinions to the robot and the user’s interest in the topic increases the motivation for exchanging opinions.

### 3.1 Condition

We investigated the above hypotheses by preparing various opinion items and asking participants to evaluate whether they believe that the robot can evaluate the target opinion and whether they want to talk with the robot about the target opinion. There are some studies in HRI (Human-Robot Interaction) that use video evaluations (Woods et al., 2006; Maeda et al., 2021). A paper showed that video and real evaluations can be similar (Woods et al., 2006). The result confirms the moderate to high agreement between the evaluation of the robot in the video and one in the real environment. In the studies of mental state attribution, a majority of paper used some kind of robot representation as the materials presented to participants (Thellman et al., 2022). It also reported that video stimuli are the second most common type of the representation. This study also evaluates the robots by video following the previous study that directly focuses on the effect of opinion attribution for dialogue motivation (Uchida et al., 2019).

**FIGURE 1:**
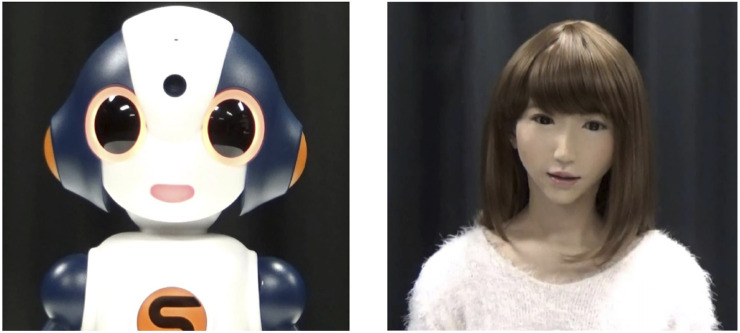
Screenshot of videos for introducing robots (left: small robot, right: android).

We prepared two conditions, one for the evaluation of the android and the other for the small robot. The participants in this experiment evaluated both conditions. This enables each participant to adopt their own same criteria for attributing opinions to the robots and for evaluating the topics on which they would like to exchange opinions. The order in which they watched the robot video was counterbalanced. Since each participant might imagine different things of robots in written instructions, we controlled their background knowledge by showing videos. We showed the participants an introductory video for each robot (small robot: https://youtu.be/5SD8PL9rRgo, android: https://youtu.be/j1RJsN3p4JY) to control the participants’ knowledge of the robots and to inform them that they are dialogue robots. The screen shots of the android and the robot are shown in Figure 1.

In the videos, the android says “I am an android,” and the small robot says “I am a robot.” The duration of each video is 5 s. The robots only said the above script such that the script would not affect the evaluation of the opinion attribution to them. The android has a extremely humanlike appearance and 44 degrees of freedom (DOF). Its lip, head, and torso movements are automatically generated from its voice using the systems developed in previous studies (Ishi et al., 2012; Sakai et al., 2016). It also expresses eye blinking at random timing. The small robot has a table-top size and 8 DOF. Its on/off of the LED at the mouth expressed the lip movement synchronized with the voice. This was realized using the function that is installed by default in it.

### 3.2 Procedure

The participants watch one of the robot’s videos, and then answer a preliminary questionnaire and a main questionnaire for the robot. They repeated this procedure for the other robot. In the preliminary questionnaire, the participants answer the questions below to evaluate the knowledge and impression of the robots.

• preQ1 (Knowledge): “Do you know the android/robot you just saw in the video?” (I know/I do not know)
• preQ2 (Age): “How old do you think the android/robot you just saw in the video is?”
• preQ3 (Interest in robots): “How interested are you in the android/robot you just saw in the video?” (0. not interested, 1. slightly interested, 2. Interested, and 3. Very interested)
• preQ4 (Preference): “How much did you like the android/robot you just saw in the video?” (0. I did not like it, 1. I liked it a little, 2. I liked it, and 3. I liked it a lot)

For each robot, participants choose one option for each question. The numbers of the choices in preQ3 and preQ4 are used to score the ratings in the analysis. We prepared preQ3 and preQ4 referring to a previous study to evaluate robots’ impression (Matsumoto, 2021).

In the main questionnaire, if they evaluate whether they attribute a specific opinion (e.g., “curry is delicious”) to the robots and whether they want to talk with it, it is possible that their motivation to exchange opinions depends not only on whether the opinion can be attributed but also on whether they agree or disagree with the opinion. In this experiment, we asked participants to evaluate whether the robot can judge each opinion (e.g., “taste of food”) and whether they want to talk with the robot about it. We prepared 100 opinions, such as “taste of food,” “beauty of paintings,” and “difficulty of mathematics.” We defined this target of opinion as a “topic.” Following the definition of subjective opinion (evaluation of a target), each opinion was created from a combination of a topic (e.g., food) and a noun-form of adjective (e.g., deliciousness of food). The topics were selected from a list of 100 topics in a previous study (Yamauchi et al., 2013), and one adjective from a list of 100 adjectives for the topic (Mizukami, 2014).

For each opinion, the participant evaluated whether they thought the robot could judge it or whether they wanted to talk about it with the robot. Since the motivation to exchange opinions is also affected by their interest in the topic, we also asked them to evaluate how interested they were in the topic. To evaluate this, we used the following questions.

• Q1 (Motivation to exchange opinions): “On each topic, how much do you want to exchange opinions with the android/robot you just saw in the video?” (0. I do not agree so, 1. I slightly agree, 2. I agree, and 3. I extremely agree)
• Q2 (Opinion attribution): “For each topic, do you think the android/robot you just saw in the video can judge it? E.g.,) Regarding “Interestingness of TV programs,” please answer “I do not agree” if you think the android cannot judge whether the TV program is interesting or not, and “I slightly agree,” “I agree,” or “I extremely agree” if you think it can with its degree.” (0. I do not agree, 1. I slightly agree, 2. I agree, and 3. I extremely agree)
• Q3 (Interest in topic): “How much interest do you have in each topic?” (0. not interested, 1. slightly interested, 2. Interested, and 3. Extremely interested)

For each topic, participants choose one of the four options for each question. The numbers of the choices in the questions are used to score the ratings in the analysis. Regarding the options of the answers, Likert scale includes the option “neither”, which causes unclear mapping of an intermediate category in this experiment. For this reason, we adopted these options above.

## 4 Result

Ninety-five Japanese people (48 males and 47 females, mean age of 24.747 years old, and variance of 6.041) participated in this experiment. We used crowdsourcing to recruit them.

### 4.1 Knowledge and impression

We evaluated the knowledge and the impression of the robots in the preliminary questionnaire. 18.947% and 30.526% of the participants knew the android and the small robot, respectively. The participants’ knowledge of the robots is considered to bias the results of the experiment. In the following, therefore, we limit the analysis to 58 participants (30 males and 28 females, mean age of 24.638 years old, and variance of 5.679) who do not know either robot. The results of the other items in the preliminary questionnaire are shown in Table 1.

**TABLE 1:** Knowledge and impression of robots.

Item	Small robot		Android	
	Mean (Variance)		Mean (Variance)	
Age	12.672	(41.013)	23.948	(17.601)
Interest in robot	0.948	(0.532)	1.172	(0.832)
Preference	1.328	(0.565)	0.931	(0.512)

### 4.2 Relationship between motivation to exchange opinions and interest in topic/opinion attribution

**FIGURE 2:**
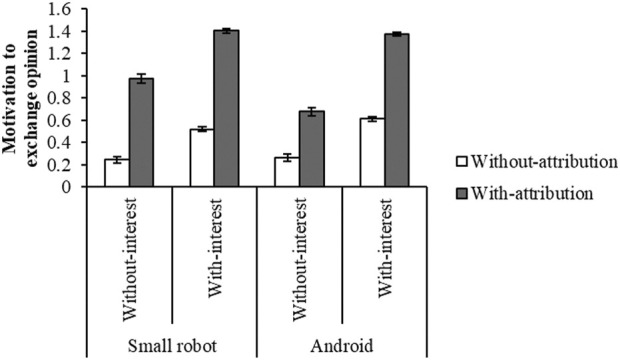
Motivation to exchange opinions for each condition. The bars show standard error.

We investigated the effects of the opinion attribution to the robots and the interest for the motivation to exchange opinions. Regarding the opinion attribution (Q2), we divided the data into two groups: those with attribution group (”1. I slightly agree, 2. I agree, and 3. I extremely agree”) and without attribution group (”0. I do not agree”). 5,724 items were evaluated without attribution and 5,876 were with attribution, and thus the percentage of items evaluated with attribution was 50.655%. Similarly, regarding the interest in the topic (Q3), we divided the data into two groups: with interest group (”1. slightly interested, 2. Interested, 3. Extremely interested”) and without interest group (”0. not interested”). Figure 2 shows the mean and standard error of motivation to exchange opinions for each condition.

A three-way ANOVA (robot × interest in topic × opinion attribution) revealed that there were significant main effects of the robot (*F* (1, 11,592) = 7.859, *p* = 0.005), the interest in topic (*F* (1, 11,592) = 498.541, *p* < 0.001), and the opinion attribution (*F* (1, 11,592) = 1,262.587, *p* < 0.001). It also did that there were significant two-way interactions of the robot × the interest in topic (*F* (1, 11,592) = 19.220, *p* < 0.001), the robot × the opinion attribution (*F* (1, 11,592) = 30.188, *p* < 0.001), the interest in topic × the opinion attribution (*F* (1, 11,592) = 40.812, *p* < 0.001). It also showed that there was a significant three-way interaction of the robot × the interest in topic × the opinion attribution (*F* (1, 11,592) = 6.117, *p* = 0.013) as shown in Table 2. It was conducted by using a statistical software HAD (Shimizu, 2016).

**TABLE 2:** Analysis of variance (*: *p* <0.05,**: *p* < 0.01).

Factor	F	p
Robot^**^	7.859	0.005
Interest in topic^**^	498.541	< 0.001
Opinion attribution^**^	1,262.587	< 0.001
Robot × Interest in topic^**^	19.220	< 0.001
Robot × Opinion attribution^**^	30.188	< 0.001
Interest in topic × Opinion attribution^**^	40.812	< 0.001
Robot × Interest in topic × Opinion attribution^*^	6.117	0.013

We conducted a simple interaction effect test since the three-way interaction was significant. It revealed that there were significant simple interaction effects on the robot × the interest in topic in the with-attribution condition (*F* (1, 11,592) = 21.380, *p* < 0.001). We conducted a simple-simple main effect test since the simple interaction effect was significant. It revealed that there were significant simple-simple main effects on the opinion attribution in the small robot and without-interest condition (*F* (1, 11,592) = 225.866, *p* < 0.001), in the small robot and with-interest condition (*F* (1, 11,592) = 1,002.086, *p* < 0.001), in the android and without-interest condition (*F* (1, 11,592) = 77.061, *p* < 0.001), and in the android and with-interest condition (*F* (1, 11,592) = 732.093, *p* < 0.001).

Another simple interaction effect test revealed that there were significant simple interaction effects on the robot × the opinion attribution both in the without-interest condition (*F* (1, 11,592) = 21.307, *p* < 0.001) and the with-interest condition (*F* (1, 11,592) = 8.943, *p* = 0.003). It revealed that there were significant simple-simple main effects on the interest in the small robot and without-attribution condition (*F* (1, 11,592) = 58.846, *p* < 0.001), in the small robot and with-attribution condition (*F* (1, 11,592) = 100.192, *p* < 0.001), in the android and without-attribution condition (*F* (1, 11,592) = 82.584, *p* < 0.001), and in the android and with-attribution condition (*F* (1, 11,592) = 314.164, *p* < 0.001).

Another simple interaction effect test revealed that there were significant simple interaction effects on the interest in topic × the opinion attribution both in the small robot condition (*F* (1, 11,592) = 7.558, *p* = 0.006) and the android condition (*F* (1, 11,592) = 39.829, *p* < 0.001). It revealed that there were significant simple-simple main effects on the robot in the without-interest and with-attribution condition (*F* (1, 11,592) = 33.256, *p* < 0.001) and in the with-interest and without-attribution condition (*F* (1, 11,592) = 9.663, *p* = 0.002).

These results suggest that not only the user’s high interest in the topic but also the opinion attribution to the robot improves the motivation to exchange opinions regardless of the robot type. Moreover, the android significantly increased the motivation when they were interested in the topic and did not attribute opinions, while the small robot significantly increased it when they were not interested and attributed opinions.

### 4.3 Motivation to exchange opinions and opinion attribution for each robot and topic field

We also analyzed the motivation to exchange opinions and the opinion attribution not only by the robot type but also by the topic field. The field is categorized by (Yamauchi et al., 2013) and listed in the Supplementary Appendix of this paper. Table 3 shows the mean and the variance of the motivation for each robot by topic field and total. The scale of the score is [0, 3]. A paired *t*-test on the mean of the motivation between robots in each topic field revealed that there is significant difference in the following topic fields: “Science” (*t* (173) = −3.894, *p* < 0.001), “Humans/Creatures” (*t* (289) = 2.701, *p* = 0.007), “Art/Hobby” (*t* (753) = 2.764, *p* = 0.006), “Industry”(*t* (463) = −5.572, *p* < 0.001), “Relationship” (*t* (463) = 5.841, *p* < 0.001), “Life”(*t* (927) = 5.421,*p* < 0.001), “Politics”(*t* (405) = −2.717, *p* = 0.007), “Communication/Computer” (*t* (115) = −2.492, *p* = 0.014), and “Culture” (*t* (579) = 2.929, *p* = 0.004). This result suggests that people are willing to exchange opinions more with the android in Humans/Creatures (88–92), Art/Hobby (41–53), Relationship (27–34), Life (11–26), and Culture (1–10), while they do more with the small robot in Science (98–100), Industry (69–76), Politics (81–87), and Communication/Computer (59–60). The numbers in the parentheses next to the topic field describes the opinion number in the Supplementary Appendix.

**TABLE 3:** Motivation to exchange opinions for each robot and topic field (*: *p* <0.05,**: *p* <0.01).

	Android Mean (Variance)	Small robot Mean (Variance)	*df*	*t*	*p*
Science^**^	0.868 (1.075)	1.230 (1.253)	173	−3.894	< 0.001
Humans/Creatures^**^	0.990 (1.256)	0.793 (1.002)	289	2.701	0.007
Media	0.888 (0.987)	0.940 (1.135)	115	−0.508	0.619
School/Study	0.770 (0.961)	0.822 (0.942)	347	−0.805	0.422
Economy/Consumption	0.892 (0.995)	0.856 (1.022)	463	0.705	0.481
Art/Hobby^**^	0.861 (1.044)	0.749 (0.916)	753	2.764	0.006
Industry^**^	0.483 (0.613)	0.716 (0.956)	463	−5.572	< 0.001
Nature	0.959 (1.016)	1.041 (1.189)	289	−1.232	0.219
Society	0.961 (1.102)	0.909 (1.087)	231	0.650	0.516
Religion/Festival	0.767 (0.963)	0.621 (0.777)	115	1.723	0.088
Relationship^**^	1.175 (1.129)	0.845 (1.021)	463	5.841	< 0.001
Life^**^	1.029 (1.129)	0.810 (0.969)	927	5.421	< 0.001
Politics^**^	0.611 (0.900)	0.756 (0.985)	405	−2.717	0.007
Communication/Computer^*^	1.095 (1.095)	1.405 (1.443)	115	−2.492	0.014
Culture^**^	1.026 (1.072)	0.886 (0.930)	579	2.929	0.004
History	0.862 (0.998)	0.948 (0.962)	57	−0.778	0.440

Table 4 lists the mean and the variance of the opinion attribution for each robot by topic field. The scale of the score is [0, 3]. A paired *t*-test on the mean of the opinion attribution between robots in each topic field revealed that there is a significant difference in the following topic fields: “Humans/Creatures” (*t* (289) = 5.797, *p* < 0.001), “Art/Hobby” (*t* (753) = 3.035, *p* = 0.002), “Industry” (*t* (463) = −2.024, *p* = 0.044), “Relationship” (*t* (463) = 7.478, *p* < 0.001), “Life” (*t* (927) = 7.057, *p* < 0.001), and “Culture” (*t* (579) = 4.507, *p* < 0.001). This result suggests that people attribute opinions more with the android in Humans/Creatures (88–92), Art/Hobby (41–53), Relationship (27–34), Life (11–26), and Culture (1–10), while they do more with the small robot in Industry (69–76). The numbers in the parentheses next to the topic field describes the opinion number in the Supplementary Appendix.

**TABLE 4:** Opinion attribution for each robot and topic field (*: *p* < 0.05, **: *p* < 0.01).

	Android mean (variance)	Small robot mean (variance)	*df*	*t*	*p*
Science	1.299 (1.321)	1.345 (1.510)	173	−0.538	0.591
Humans/Creatures^**^	0.952 (1.008)	0.593 (0.720)	289	5.797	< 0.001
Media	1.293 (1.252)	1.112 (1.248)	115	1.72	0.088
School/Study	0.934 (1.117)	0.928 (1.162)	347	0.105	0.916
Economy/Consumption	1.019 (1.073)	0.974 (1.313)	463	0.873	0.383
Art/Hobby^**^	0.817 (0.947)	0.712 (0.928)	753	3.035	0.002
Industry^*^	0.879 (0.944)	0.981 (1.168)	463	−2.024	0.044
Nature	1.100 (1.142)	1.069 (1.289)	289	0.510	0.611
Society	1.047 (1.093)	0.944 (1.205)	231	1.528	0.128
Religion/Festival	0.647 (0.683)	0.552 (0.632)	115	1.257	0.211
Relationship^**^	0.944 (1.098)	0.606 (0.814)	463	7.478	< 0.001
Life^**^	0.853 (0.988)	0.622 (0.896)	927	7.057	< 0.001
Politics	0.894 (1.009)	0.919 (1.102)	405	−0.490	0.625
Communication/Computer	1.759 (1.263)	1.871 (1.453)	115	−0.894	0.373
Culture^**^	0.910 (0.887)	0.717 (0.835)	579	4.507	< 0.001
History	0.810 (0.893)	0.741 (0.967)	57	0.600	0.551

## 5 Discussion

In this study, we hypothesized that opinion attribution affects the user’s motivation to exchange opinions with humanoid robots. The experimental result suggests that this attribution improve their motivation. Moreover, approximately half of the subjective opinions were attributed to the humanoid robots. According to development of robotics and AI technologies, there is a possibility that this degree will improve. Another analysis revealed that the android significantly increased the motivation when they were interested in the topic and did not attribute opinions, while the small robot significantly increased it when they were not interested and attributed opinions. The results of this experiment supported the hypothesis and suggested that the importance of opinion attribution can be generalized to humanoid robots. The contributions of this study are that we clarified the influence of opinion attribution to humanoid robots on users’ motivation to exchange opinions with them, and clarified the topics that improve the user’s motivation to exchange opinions with each type of humanoid robot. While interest in a topic is a self-oriented factor formed by one’s past experiences, opinion attribution is an other-oriented factor formed by the conversation partner (here, the android and the small robot). Based on the findings, both self-oriented and other-oriented factors must be considered when designing dialogue robots that stimulate the user’s motivation to exchange opinions.

There are many dialogue systems that refer to human-human dialogue datasets (e.g., (Huang et al., 2022)) to generate the system’s utterance. These datasets would also contains utterance data including subjective opinions that cannot be attributed to the robot. Based on the results of this study, when implementing a dialogue system on a robot, it is necessary to consider not only the linguistic connection of utterances, but also whether the subjective opinions can be attributed to the robot. To avoid this problem, it can take a strategy to avoid discussing topics that the user cannot attribute opinions to. However, considering the robots’ ability, there are few items that can be attributed to robots. This means that the subjective opinions which are exchanged between the user and robot are extremely limited. Even if the robot told us that it had excellent coffee, we would not believe that it understands the taste of coffee. However, we frequently talk about a topic such as the taste of coffee, and the limitation would make the user lose their motivation to exchange opinions with the robot. To stimulate the user’s motivation regarding any topic, it is necessary to make the robot imagine that it can judge the target topic (i.e., the user can attribute the opinion to the robot).

Moreover, we investigated the effect of the robot type on the motivation to exchange opinions. The android significantly increased the motivation when they were interested in the topic and did not attribute opinions, while the small robot significantly increased it when they were not interested and attributed opinions. We can think of situations in which people are interested and do not attribute opinions, such as chatting in which they talk about casual things. Androids could be useful in such interactions. There are also situations in which they are not interested and attribute opinions such as listening to a lecture they are not interested in. The small robot may be useful in such educational situations. Also, the fact that people want to exchange opinions with androids if they are interested in the topic, even if they do not attribute opinions to it, shows the usefulness of androids as dialogue robots.

In this study, we further analyzed the motivation and the opinion attribution between robots for each topic field. The scores for humans/creatures, art/hobby, relationship, life, and culture were significantly higher for the android. Many of them are related to humans and their society. This may be because the appearance of the android closely resembles a human. However, the scores for science, industry, politics, and communication/computer were significantly higher for the small robot. They were mostly related to science and technology. This may be attributed to the non-human appearance of the small robot. We examine the results from the viewpoint of whether they exceed 1 since “disagree” is scored as 0 and “slightly agree” is scored as 1 in the questionnaire. For both robots, the mean scores were above 1 only for communication/computer. This probably because robots is an embodiment of information technology. The scores of the motivation did not exceed 2 for any of the topic fields (see Table 3), which indicates that the motivation is not high. This can be because the interaction was limited to the short video observation, and thus the interaction was not enough to arouse the desire for exchanging opinions. The Uncanny Valley (Mori et al., 2012) is a phenomenon that extremely human-like artificial entities can be uncanny. Research on this phenomenon investigates familiarity, which is not consistent with the motivation to exchange opinions targeted in this study. On the other hand, it is meaningful to discuss the relationship with this phenomenon. The results of this study on the motivation of each robot indicate that in some cases the android is significantly higher, and in other cases the small robot is. Thus, it may be difficult to explain the motivation based solely on human-likeness of the robots. Also, it should be noted that there is a case in which this phenomenon cannot be applied an android (Bartneck et al., 2009).

Subsequently, we discuss the opinion attribution between robots for each topic field. In the topic fields of humans/creatures, art/hobby, relationship, life, and culture, more subjective opinions were attributed to the android than to the small robot, while in the industry, more were attributed to the small robot. This can be similar to the results for the motivation, and the reason can also be similar. Many previous studies have shown that the more human-like robots become, the more they are attributed mental state (Krach et al., 2008; Broadbent et al., 2013; Takahashi et al., 2014; Martini et al., 2016; 2015; Xu and Sar, 2018; Banks, 2020; Manzi et al., 2020). Although opinions are considered to be a part of mental state, the results of this study suggest that people attribute opinions to smaller robots more than to more human-like androids in some topics. This may indicate that topics and task should be considered in the attribution issue. For both robots, the topics with the mean score above 1 were science, media, nature, and communication/computer. This can derive from the characteristics of robots themselves, as well as the reason discussed in the motivation. The topics that are not above 1 for the motivation but for the opinion attribution were science, media and nature. Although these topic fields have a relatively high degree of opinion attribution to the robots, they may not stimulate a desire for dialogue because the exchange of opinions related to them can be limited to specific groups such as experts in the fields. The scores of the attribution did not exceed 2 for any of the topic fields (see Table 4), which indicates that the attribution is not high. This can also stem from the short video observation.

Here, we discuss the relationship between robots’ appearance (embodiment) and intelligence. The degree of opinion attribution is greatly influenced by the degree of development of the technologies of robots and AI. The results of this study revealed that the degree of opinion attribution differs depending on the type of robot because they look different. The attribution of opinions (i.e., the ability to make judgments) may indicate that appearance influences intelligence. Previous studies also reported that the appearance of robots and agents affects the impressions of humans (Uchida et al., 2017b). The results of this study may contribute to the clarification of the relationship between them. Since not only appearance but also voice may have an influence, various modalities of communication should be investigated. It is also necessary to examine how opinion attribution is affected by the presence or absence of a body, for example, by comparing a robot in front of the user with a virtual agent (e.g., (Kidd and Breazeal, 2004; Powers et al., 2007)) or a text chatbot system (e.g., (Shuster et al., 2022)).

Finally, we address the limitations of this study. This study also evaluated the robots by video to follow the previous study (Uchida et al., 2019). The results may differ between a video evaluation and an evaluation by actually seeing the robots. Video provides less information regarding the robots’ modality, which may change the results. As the android has embodiment that closely resembles humans and can interact with various modalities (e.g., gestures), opinion attribution and motivation to exchange opinions may change through interaction. We used an android, which is a humanoid robot that closely resembles a human in appearance, and a small robot that has a machine-like appearance. This means that not all humanoid robots could be evaluated. Moreover, although significant differences were found in some evaluations, the absolute values are not large. To solve this problem, we need to design the interaction between the robot and the user. It is also necessary to examine how the evaluation changes when the robots interact or perform tasks, rather than just introducing themselves unilaterally as in this case. Investigating the relationship between opinion attribution and motivation to exchange opinions with the robots through interaction will also be necessary in future research. Though we adopted a topic classification used in the previous study (Yamauchi et al., 2013), there can be multiple variations in the classification depending on the participants. Also, what we defined as an opinion is not only a topic but also a set of a noun-form of adjective. Therefore, a systematic investigation of the types of adjectives is necessary. Furthermore, the results of this experiment may vary depending on the groups of the participants. Specifically, the results of the experiment may vary depending on the attributes of the participants (e.g., occupation and culture) and their knowledge of the robot. Future studies must include their attribution and the degree of involvement with the robot in daily life.

## 6 Conclusion

This study explored how the user’s motivation to exchange opinions with the humanoid robots is influenced by attributions of opinions to them. We examined the effect by preparing various opinions of two kinds of humanoid robots. The experimental result suggests that not only the users’ interest in the topic but the attribution of the subjective opinions to them influence their motivation to exchange opinions. Another analysis revealed that the android significantly increased the motivation when they were interested in the topic and did not attribute opinions, while the small robot significantly increased it when they were not interested and attributed opinions.

## Data Availability

The raw data supporting the conclusion of this article will be made available by the authors, without undue reservation.

## Footnotes

1
https://www.vstone.co.jp/products/sota/
