# Supplementary material for: Opinion attribution improves motivation to exchange subjective opinions with humanoid robots
Source: Front Robot AI. 2024 Feb 19;11:1175879. doi: 10.3389/frobt.2024.1175879 (PMC10909954; doi:10.3389/frobt.2024.1175879)
Supplement: Supplementary file 1 [file DataSheet1.pdf]

Supplementary Material

The items of the opinions used in this experiment are listed below. The format of description is "[Noun-form of adjective] of [Topic] (Topic field, Higher adjective classification, Lower adjective classification)."

1. Taste of food (Culture, Attribution, Object)
2. Taste of alcohol (Culture, Attribution, Object)
3. Fashion of clothes (Culture, Attribution, Object)
4. Joy of travel (Culture, Emotion, Emotion)
5. Difficulty of sports (Culture, Attribution, Things)
6. Comfort of home (Culture, Attribution, Object)
7. Difficulty of language (Culture, Attribution, Things)
8. Interest of publication (Culture, Emotion, Emotion)
9. Goodness of season (Culture, Attribution, Things)
10. Uniqueness of culture (Culture, Attribution, Things)
11. Cleanliness of town (Life, Attribution, Object)
12. Nostalgia of home (Life, Emotion, Emotion)
13. Inconvenience of traffic (Life, Attribution, Things)
14. Richness of everyday life (Life, Attribution, Things)
15. Convenience of machine (Life, Attribution, Object)
16. Cleanliness of housekeeping (Life, Attribution, Things)
17. Joy of party (Life, Emotion, Emotion)
18. Hardness of moving house (Life, Attribution, Things)
19. Difficulty of procedure (Life, Attribution, Things)
20. Sadness of love (Life, Emotion, Emotion)
21. Joy of marriage (Life, Emotion, Emotion)
22. Difficulty of childbirth and childcare (Life, Attribution, Things)
23. Vividness memory (Life, Attribution, Things)
24. Difficulty of dream and goal (Life, Attribution, Things)
25. Pain of distress (Life, Emotion, Emotion)
26. Sadness of death (Life, Emotion, Emotion)
27. Kindness of family (Relationship, Attribution, Person)
28. Importance of friend (Relationship, Attribution, Object)
29. Goodness of personality (Relationship, Attribution, Person)
30. Complexity of emotion (Relationship, Attribution, Things)
31. Beauty of appearance (Relationship, Attribution, Things)
32. Difficulty of human relationship (Relationship, Attribution, Things)
33. Pain of fight and trouble (Relationship, Emotion, Emotion)
34. Elegance of manner and habit (Relationship, Attribution, Things)
35. Fun of school (elementary, middle and high school) (School/Study, Emotion, Emotion)
36. Stylishness of school (university) (School/Study, Attribution, Object)
37. Goodness of grade (School/Study, Attribution, Things)
38. Simplicity of test (School/Study, Attribution, Things)
39. Joy of learning (School/Study, Emotion, Emotion)

40. Difficulty of research (School/Study, Attribution, Things)
41. Joy of music (Art/Hobby, Emotion, Emotion)
42. Beauty of painting (Art/Hobby, Attribution, Object)
43. Rarity of craft (Art/Hobby, Attribution, Broad)
44. Vividness of photo (Art/Hobby, Attribution, Object)
45. Goodness of film and theater (Art/Hobby, Attribution, Object)
46. Importance of performing art (Art/Hobby, Attribution, Things)
47. Goodness of art (Art/Hobby, Attribution, Things)
48. Joy of hobby (Art/Hobby, Emotion, Emotion)
49. Expensiveness of collection (Art/Hobby, Attribution, Object)
50. Goodness of Sunday carpenter (Art/Hobby, Attribution, Things)
51. Joy of handicraft (Art/Hobby, Emotion, Emotion)
52. Joy of Gambling (Art/Hobby, Emotion, Emotion)
53. Goodness of playing and game (Art/Hobby, Attribution, Things)
54. Complexity of religion (Religion/Festival, Attribution, Things)
55. Joy of festival (Religion/Festival, Emotion, Emotion)
56. Interest of history (History, Emotion, Emotion)
57. Correctness of media (Media, Attribution, Things)
58. Popularity of celebrity (Media, Attribution, Person)
59. Accuracy of communication (Communication/Computer, Attribution, Things)
60. Complexity of computer (Communication/Computer, Attribution, Object)
61. Expensiveness of shopping (Economy/Consumption, Attribution, Object)
62. Hardness of work (Economy/Consumption, Attribution, Things)
63. Difficulty of finding job (Economy/Consumption, Attribution, Things)
64. Newness of business (Economy/Consumption, Attribution, Things)
65. Danger of stock (Economy/Consumption, Attribution, Broad)
66. Wealth of economy (Economy/Consumption, Attribution, Things)
67. Difficulty of international economy (Economy/Consumption, Attribution, Things)
68. Pain of tax (Economy/Consumption, Emotion, Emotion)
69. Importance of industry (Industry, Attribution, Things)
70. Beauty of car (Industry, Attribution, Object)
71. Importance of heavy industry (Industry, Attribution, Things)
72. Precision in machinery industry (Industry, Attribution, Things)
73. Danger of construction and civil engineering (Industry, Attribution, Broad)
74. Richness of energy (Industry, Attribution, Things)
75. Safety of agriculture and forestry (Industry, Attribution, Things)
76. Valuableness of fishery Industry (Industry, Attribution, Things)
77. Fear of incident and accident (Society, Emotion, Emotion)
78. Fear of discrimination (Society, Emotion, Emotion)
79. Rapidness of aging population with low birthrate (Society, Attribution, Things)
80. Richness of social security and welfare (Society, Attribution, Things)
81. Fairness of politics (Politics, Attribution, Things)
82. Difficulty of law (Politics, Attribution, Things)
83. Intensity of social movement (Politics, Attribution, Things)
84. Superiority of election (Politics, Attribution, Things)

85. Advantage of diplomacy (Politics, Attribution, Things)
86. Fear of war (Politics, Emotion, Emotion)
87. Importance of meeting (Politics, Attribution, Things)
88. Pain of body (Humans/Creatures, Emotion, Perception)
89. Seriousness of healthcare (Humans/Creatures, Attribution, Things)
90. Flashy of beauty (Humans/Creatures, Attribution, Things)
91. Cuteness of animal (Humans/Creatures, Attribution, Object)
92. Cleanliness of plant (Humans/Creatures, Attribution, Object)
93. Warmth of weather (Nature, Attribution, Things)
94. Beauty of nature (Nature, Attribution, Object)
95. Magnitude of disaster (Nature, Attribution, Things)
96. Difficulty of environmental issue (Nature, Attribution, Things)
97. Wonder of Universe (Nature, Attribution, Object)
98. Difficulty of math (Science, Attribution, Things)
99. Difficulty of science (Science, Attribution, Things)
100. Importance of technology (Science, Attribution, Things)
